# Metabolites from Marine-Derived Fungi as Potential Antimicrobial Adjuvants

**DOI:** 10.3390/md19090475

**Published:** 2021-08-25

**Authors:** Fernando Durães, Nikoletta Szemerédi, Decha Kumla, Madalena Pinto, Anake Kijjoa, Gabriella Spengler, Emília Sousa

**Affiliations:** 1 Laboratory of Organic and Pharmaceutical Chemistry, Department of Chemical Sciences, Faculty of Pharmacy, University of Porto, Rua de Jorge Viterbo Ferreira, 228, 4050-313 Porto, Portugal; fduraes5@gmail.com (F.D.); madalena@ff.up.pt (M.P.); 2 CIIMAR–Interdisciplinary Centre of Marine and Environmental Research, University of Porto, Novo Edifício do Terminal de Cruzeiros do Porto de Leixões, Avenida General Norton de Matos, S/N, 4450-208 Matosinhos, Portugal; Decha1987@hotmail.com (D.K.); ankijjoa@icbas.up.pt (A.K.); 3 Department of Medical Microbiology, Albert Szent-Györgyi Health Center, Faculty of Medicine, University of Szeged, Semmelweis utca 6, 6725 Szeged, Hungary; szemeredi.nikoletta@med.u-szeged.hu; 4 ICBAS–Institute of Biomedical Sciences Abel Salazar, Universidade do Porto, Rua de Jorge Viterbo Ferreira 228, 4050-313 Porto, Portugal

**Keywords:** marine-derived fungal metabolites, antimicrobial activity, efflux pump inhibition, biofilm inhibition, quorum-sensing inhibition

## Abstract

Marine-derived fungi constitute an interesting source of bioactive compounds, several of which exhibit antibacterial activity. These acquire special importance, considering that antimicrobial resistance is becoming more widespread. The overexpression of efflux pumps, capable of expelling antimicrobials out of bacterial cells, is one of the most worrisome mechanisms. There has been an ongoing effort to find not only new antimicrobials, but also compounds that can block resistance mechanisms which can be used in combination with approved antimicrobial drugs. In this work, a library of nineteen marine natural products, isolated from marine-derived fungi of the genera *Neosartorya* and *Aspergillus*, was evaluated for their potential as bacterial efflux pump inhibitors as well as the antimicrobial-related mechanisms, such as inhibition of biofilm formation and quorum-sensing. Docking studies were performed to predict their efflux pump action. These compounds were also tested for their cytotoxicity in mouse fibroblast cell line NIH/3T3. The results obtained suggest that the marine-derived fungal metabolites are a promising source of compounds with potential to revert antimicrobial resistance and serve as an inspiration for the synthesis of new antimicrobial drugs.

## 1. Introduction

Nature has been one of the main sources of bioactive compounds, either as an inspiration for synthesis or semi-synthesis, or directly used, through isolation from natural matrices. Even currently, natural products play an important role in therapeutics, as well as in agriculture and aquaculture [1]. Despite significant advancements in this field, the potential of natural products is still underexplored [2].

Developments in technology and methodologies have allowed more effective prospection of the marine environment and the determination of chemical and biological profiles of compounds derived from marine sources. As a result, new scaffolds have been discovered, leading to research focusing on these topics, with compounds derived from marine macroorganisms having been already approved for therapeutics [2,3]. However, in recent years, the focus has shifted towards marine microorganisms, such as bacteria and fungi, which have the advantage of being easily cultivated in a laboratory setting and with a capacity to produce larger amounts of secondary metabolites [2]. Furthermore, as marine microorganisms have been unexplored until recently, several new scaffolds [4], with application in several major diseases, especially infectious diseases, are being discovered [5,6].

Marine-derived fungi constitute an important and prolific source of secondary metabolites that are useful for development of anti-infective agents. Compounds isolated from marine-derived fungi include alkaloids, polyketides, coumarins, polyphenols, acetophenones, and xanthones, among others [1,3,7,8,9]. Specifically, the *Aspergillus* and *Neosartorya* genera have been extensively studied and recognized for the bioactive compounds they are able to produce [10,11,12,13,14,15]. Our group has been studying the potential of compounds isolated from these marine-derived fungi as potential antimicrobial agents, some of which have shown antibacterial activity against susceptible and resistant strains of clinically relevant bacteria [16,17,18,19].

Currently, one of the main quests is the search for new compounds that not only exhibit antimicrobial activity, but also can potentiate the antimicrobial activity and revert a resistance to antibiotics already in therapeutics [20]. Thus, in the present work, the ability of compounds, isolated from marine-derived fungi of the *Neosartorya* species and *Aspergillus elegans*, to revert antimicrobial resistance mechanisms was studied.

The mechanisms that lead to antimicrobial resistance can manifest in different ways including the overexpression of efflux pumps, ubiquitous transmembrane transporters that can secrete antimicrobials outside the cell or to the periplasm [21,22,23,24]. Although there are some studies on marine natural products that have been proven useful to circumvent multidrug resistance in the mammalian efflux pump P-glycoprotein [25], reports on bacterial efflux pumps are sparse [26,27,28]. Thus, it is urgent to find new molecules with potential to serve as antimicrobial adjuvants, with the advantage of not being prone to resistance [29].

In our pursuit for antibacterial compounds against multidrug-resistant (MDR) bacteria from marine-derived fungi of the genera *Aspergillus* and *Neosartorya* from tropical seas, we have discovered specialized metabolites with antibacterial activity, particularly in multidrug-resistant clinical isolates. This prompted us to investigate the potential inhibition of mechanisms of bacterial resistance and virulence of the compounds from the sea, using a library of nineteen compounds, isolated from marine-derived fungi, to study their antimicrobial activity and efflux pump inhibition in relevant bacterial strains. Their potential as inhibitors of biofilm formation and quorum-sensing (QS) was also studied, as these mechanisms are closely related to the inhibition of efflux pumps [30,31,32,33].

Docking studies were performed in order to evaluate if these compounds have similar predicted binding scores to those of compounds described as bacterial efflux pump inhibitors, and also to visualize them in their targets and identify the residues with which they are interacting.

The compounds studied herein were isolated from four different strains of fungi: six compounds from a soil fungus *Neosartorya siamensis* (KUFC 6349) [16]; four compounds from a marine-derived fungus *N. takakii* (KUFC 7898), which was isolated from the marine macroalga *Amphiroa* sp. [19]; one compound from the diseased coral-derived fungus *N. laciniosa* (KUFC 7869) [34], and eight compounds from a marine-derived *Aspergillus elegans* (KUFA 0015), which was isolated from the marine sponge *Monanchora unguiculata* [17].

## 2. Results and Discussion

### 2.1. Compounds

**Figure 1:**
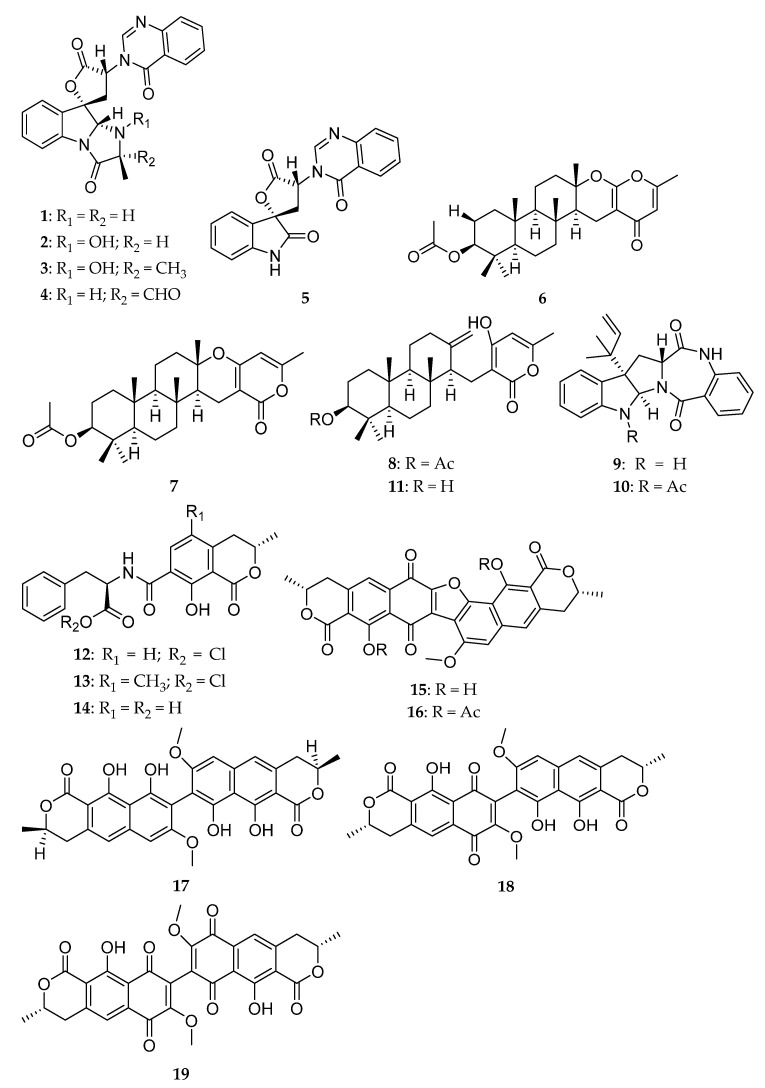
Structures of **1**–**19**.

The isolation and structure elucidation of the compounds used in this study have been previously described, and some of them have already been assayed for their antimicrobial activity. Five indole alkaloids, tryptoquivaline F (**1**), tryptoquivaline H (**2**), tryptoquivaline L (**3**), tryptoquivaline O (**4**), and 3′-(4-oxoquinazolin-3-yl)spiro(1*H*-3,5′-oxolone)-2,2′-dione (**5**), and a meroditerpene, chevalone C (**6**), were obtained from *N. siamensis* [16]. Two meroditerpenes, chevalone B (**7**) and aszonapyrone A (**8**), and two indole alkaloids, aszonalenin (**9**) and acetyl aszonalenin (**10**), were obtained from *N. takakii* [19]. A meroditerpene, aszonapyrone B (**11**), was obtained from *N. laciniosa* [34]. Finally, eight compounds including ochratoxin A (**12**) and its methyl ester derivative (**13**), ochratoxin B (**14**), the dimeric naphthopyranones rubrosulphin (**15**) and its diacetate derivative (**16**), vioxanthin (**17**), viomellein (**18**), and xanthomegnin (**19**) were obtained from *A. elegans* [17]. It is worth mentioning that **17**–**19** present a similar scaffold to that of xanthones, which were previously described as potential efflux pump inhibitors [35]. Figure 1 shows the structures of **1**–**19**.

### 2.2. Antimicrobial Activity

For the compounds to be tested for their potential as bacterial efflux pump inhibitors, their antimicrobial activity was evaluated to determine their minimum inhibitory concentration (MIC) in the strains under investigation. Although **1**–**19** were previously investigated for their antimicrobial activity in Gram-positive and Gram-negative bacteria [16,17,19], there were no reports on antimicrobial activity of these compounds in the tested strains, *Staphylococcus aureus* 272123 and the *acrA* gene deleted *Salmonella enterica* serovar Typhimurium SL1344 (SE03). As such, **1**–**19** were tested for their antibacterial activity against *S. aureus* 272123 and SE03. Table 1 shows MIC values of the tested compounds against *S. aureus* 272123 and SE03 and ciprofloxacin was used as positive controls.

Analysis of the data in Table 1 shows that none of the tested compounds are active against SE03, while **6**, **8**–**10**, **13**, **14**, and **17**–**19** displayed growth inhibition against *S. aureus* 272123. It is important to point out that **15** and **16** are structurally related to **17**–**19**. The only difference is the pyran ring between the two naphthopyranone moieties in **15** and **16**, which confers their rigid structures, was absent in **17**–**19**. These rigid structures could be responsible for the lack of antibacterial activity. In fact, **15**–**19** have already been tested for their antibacterial activity in susceptible and resistant strains, and it was found that **17**–**19** exhibited promising antibacterial activity, whereas **15** and **16** did not display any observable MIC [17]. In addition, **1**–**3**, **5**, **8**, and **13** have already been found to exhibit antibacterial activity against the American Type Culture Collection (ATCC) and multidrug-resistant strains [16,17,18].

Concerning their antimicrobial activity in *S. aureus* 272123 and SE03, structure-activity relationships (SAR) could be established for some series of compounds. For the alkaloids **9** and **10**, it is observed that a substitution of the amine in the indoline moiety leads to loss of potency in the Gram-positive model tested. In the case of the dimeric naphthopyranones **15**–**19**, substitution of the naphthodiol moiety with a naphthoquinone core, in **19**, also leads to a decrease of potency in the same model. It was also noted that a furan ring connecting the two naphthopyranone moieties leads to loss of activity, as opposed to a simple bond.

### 2.3. Efflux Pump Inhibition Assay

The ability of **1**–**19** to inhibit bacterial efflux pumps was further evaluated through the real-time ethidium bromide accumulation assay, which measures the relative fluorescence originated by ethidium bromide, an efflux pump substrate, over the time of the assay. For this purpose, two bacterial strains were used: one Gram-positive and one Gram-negative. For the Gram-positive, *S. aureus* 272123, a clinical oxacillin- and methicillin-resistant strain, was used. This strain was used to compare the activity of the isolated compounds with that of derivatives previously tested in the same system [35,36]. However, previous studies with this strain suggested that the expression of the *norA* gene, a core gene of *S. aureus*, remained unchanged [37]. Moreover, despite the NorA pump is ubiquitous for this bacterium, it is not the main pump responsible for the efflux [38]. For the study of Gram-negative efflux systems, the SE03 strain was used.

The main goal of this study was to perform the first screening of marine fungal metabolites and their ability to modulate the efflux of ethidium bromide. To preserve bacterial viability, the compounds were tested at one-third of their MIC, and the concentration of each compound is shown in Table S1. The results were expressed as relative fluorescence index (RFI), which was calculated based on the mean of the relative fluorescence units (Table S1). As positive controls, two known efflux pump inhibitors were used: reserpine and carbonyl cyanide 3-chlorophenylhydrazone (CCCP), at 25 µM (sub-MIC concentration), for *S. aureus* 272123 and SE03, respectively.

**Figure 2:**
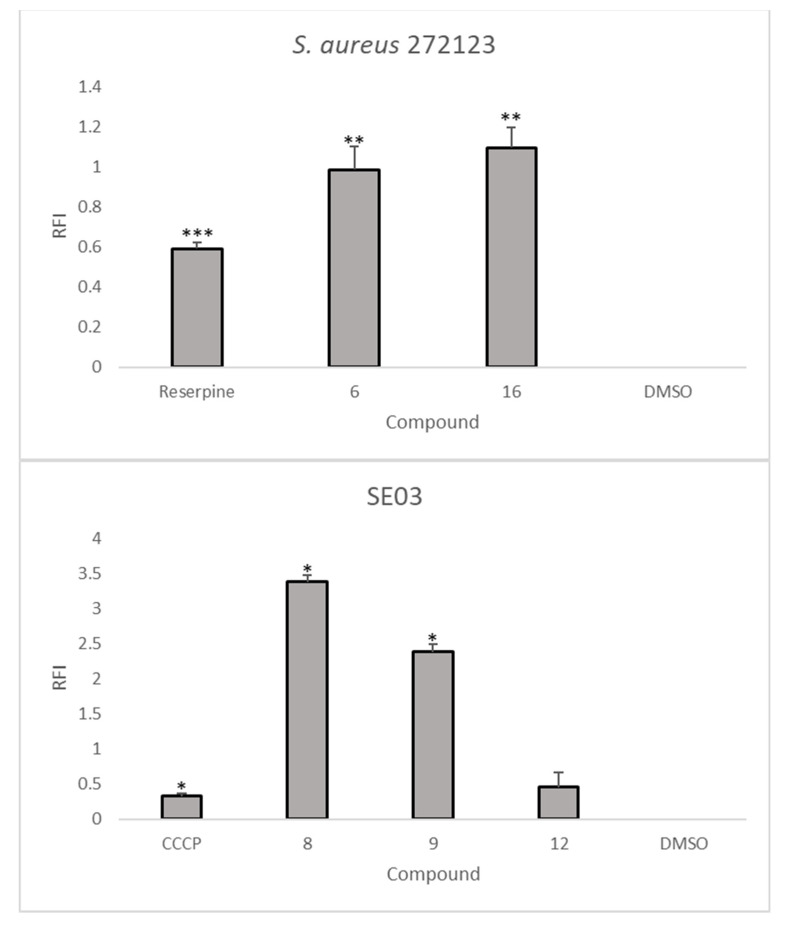
Comparison of the RFI of different compounds to those of the positive controls [*S. aureus* 272123 (top); SE03 (bottom)]. Results are presented as mean ± SD. Statistical comparisons were performed using the *t*-test [* *p* < 0.05; ** *p* < 0.01; *** *p* < 0.001 vs. control (DMSO 1% *v/v*)].

The results show that **6**, **7**, and **16** displayed higher RFI values than the positive control reserpine when tested with *S. aureus* 272123, meaning that ethidium bromide remains intracellularly and forms a fluorescent complex with DNA (Figure 2). In the same way, **8**, **9**, and **12** showed higher RFI values than CCCP in SE03 (Figure 2). Therefore, these compounds were considered effective for this purpose. However, some compounds displayed negative RFI values, i.e., they produce less fluorescence than the negative control, dimethyl sulfoxide (DMSO, 1% *v*/*v*), and were consequently considered ineffective as inhibitors of efflux pumps. However, it cannot be ruled out that the results obtained could also be due to the fluorescence emitted by the compound itself. For this purpose, the relative fluorescence of the compounds was recorded over time, with a solution of the tested compounds, a solution of ethidium bromide (EB), and a solution of the tested compounds with ethidium bromide. Accordingly, only **7** was tested, since its relative fluorescence at the starting point of the experiment was much higher than those of the controls for both bacterial strains (results not shown). The fluorescence assay showed that this compound presents an erratic curve when applied in combination with ethidium bromide (Figure S1, Supplementary Data), and the results obtained could not be considered.

The results showed that the meroditerpene **6** and the dimeric naphthopyranone **16** were able to inhibit the efflux of ethidium bromide in *S. aureus* 272123. On the other hand, the meroditerpene **8** and the alkaloid **9** showed significant inhibition of the efflux of ethidium bromide in SE03, while the amide-containing isochromone **12** showed a more modest inhibition.

However, further studies are warranted to confirm the exact mode of action through which these compounds inhibit the efflux of ethidium bromide, as other mechanisms, such as membrane depolarization, can also lead to a decrease in relative fluorescence. Nevertheless, these compounds were chosen for further assays in resistance and virulence mechanisms related to efflux pumps, along with the compounds that displayed antibacterial activity.

### 2.4. Molecular Visualization

The compounds that displayed the most promising results for the efflux pump inhibition assay, **6**, **8**, **9**, and **16**, were visualized in the most relevant bacterial efflux pumps: the AcrAB-TolC efflux system, belonging to the resistance-nodulation-division (RND) family, which acquires great importance in Gram-negative bacteria [39], and the NorA efflux pump, which is more relevant in Gram-positive bacteria, and is part of the major facilitator superfamily (MFS).

For this purpose, docking studies were performed with different components of the AcrAB-TolC efflux system, whose crystal structures are all deposited in the Protein Data Bank (AcrB: 4DX5, AcrA: 2F1M, TolC: 1EK9), and in a homology model of the NorA pump (Supplementary Data). Compounds **1**–**19** were docked into sites already described in the literature [40,41,42]. As positive controls, compounds with reported activity in the efflux systems of Gram-positive and Gram-negative bacteria were used, such as reserpine, phenyl-arginyl-β-naphthylamide (PAβN), D13-9001, doxorubicin, MBX-3132, and minocycline. The results of the docking studies are shown in Table S2 (Supplementary Data). The main findings were the higher predicted affinity to AcrB in detriment to the other components of the AcrAB-TolC efflux system, and that AcrA was predicted to display the least affinity. It also showed that the compounds would, in general, present docking scores similar to compounds described as bacterial efflux pump inhibitors.

**Figure 3:**
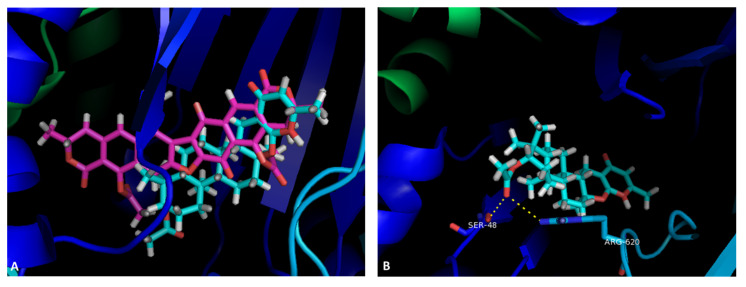

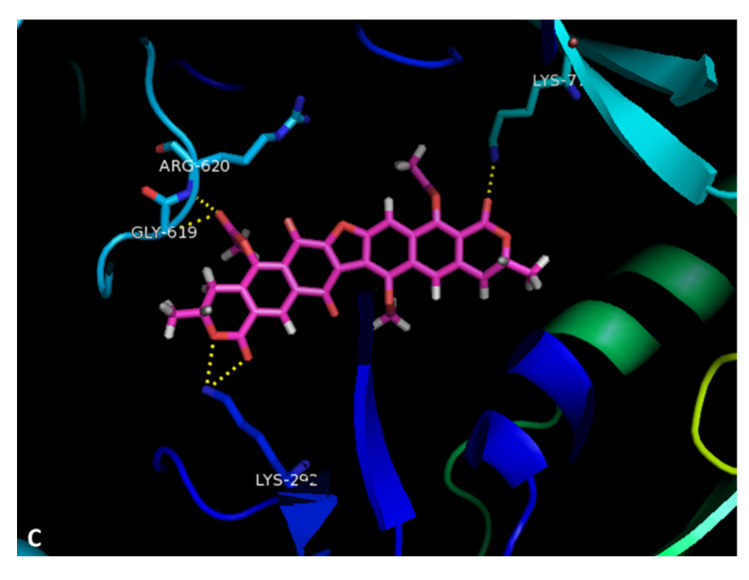
(**A**) Molecular visualization of **6** (blue) and **16** (pink) in the SBS of AcrB; (**B**) interactions of **6** with the SBS; (**C**) interactions of **16** with the SBS.

In light of these results, the binding site and the residues involved in the interactions with **6**, **8**, **9**, **12**, and **16** were investigated using PyMol. In accordance with initial docking studies (Table S2), **6** and **16** were visualized in the SBS of AcrB, and the results are shown in Figure 3. A general view shows that both compounds interact approximately at the same binding site, with a portion of **16** almost superimposed with **6** (Figure 3A). A more detailed analysis shows that **6** interacts with Ser-48 and Arg-620, being the acetyl moiety responsible for these interactions (Figure 3B). It can also establish a π-π stacking with Phe-615. Compound **16** interacts with the amine in Lys-292, through the oxygen atoms of the lactone moiety. One of the acetyl groups can also interact through a hydrogen bond with Gly-619 and Arg-620, and the carbonyl in another lactone ring also forms a hydrogen bond with Lys-770 (Figure 3C) as well as π-π stacking with Phe-615. Both compounds also presented other non-polar interactions (not shown). The interaction between the acetyl moiety and Gly-619 and Arg-620 may explain the lack of activity of **15**, which is a non-acetylated analogue of **16**. Interestingly, xanthones that were studied in the same assay also demonstrated this kind of activity and also displayed predicted interaction with Gly-619 and Arg-620 [35].

**Figure 4:**
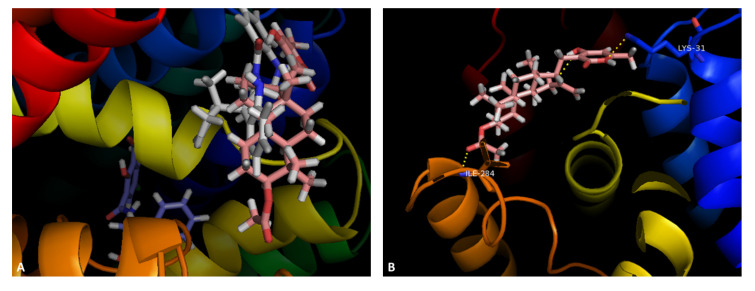

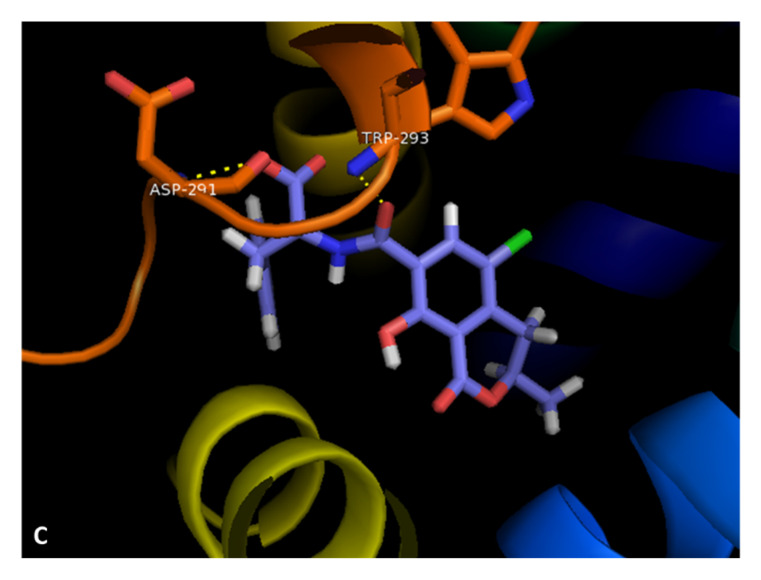
(**A**) Molecular visualization of **8** (pink), **9** (white) and **12** (blue) in the CS of the NorA homology model; (**B**) interactions of **8** with the CS; (**C**) interactions of **12** with the CS.

The same procedure was applied for the CS of the homology model of NorA and **8**, **9** and **12**. A general view of the compounds shows that **8** and **9** are predicted to bind near each other, whereas **12** is predicted to bind in a different site (Figure 4A). Compound **8** showed the capability of establishing two hydrogen bonds, one between the oxygen of the lactone ring and the nitrogen in Lys-31, and another between the carbonyl in the acetyl moiety and Ile-284 (Figure 4B). It could also establish a T-shaped π-π interaction with Tyr-225. Compound **9** did not show any predicted interactions with this method. This can be due to the use of a homology model, or the compound itself may act as a steric hinderer, making the entrance of substrates difficult, as this visualization is being performed in the cytoplasmic side of the model. Compound **12** can establish a hydrogen bond between the carboxylic acid moiety and the amine group in Asp-291, and the amide carbonyl and Trp-293 (Figure 4C). It can also establish π-π interactions with Phe-47 and Tyr292. All the compounds presented non-polar interactions with the tested macromolecules (results not shown).

### 2.5. Inhibition of Biofilm Formation and Quorum-Sensing

To gain a better insight of the full potential of the most promising compounds, all the compounds that presented antibacterial activity and/or inhibition of the ethidium bromide accumulation were tested, regardless of inhibiting the efflux in the Gram-positive or Gram-negative models. As such, compounds tested in these assays were **6**–**10**, **12**–**14**, and **16**–**19**. For the study of the inhibition of biofilm formation, the strains used were *S. aureus* ATCC 29213 and *S. aureus* 272123. Reserpine was used as a positive control, since this compound was used as a positive control in the efflux pump inhibition assay and has been described as an inhibitor of both efflux pumps and biofilm formation [43,44].

The ability of the compounds to inhibit biofilm formation, prevent adhesion, or degrade biofilm was expressed in percentage (%), and was calculated based on the mean of absorbance units. The compounds were tested at ½ MIC. As such, the MIC of the compounds were tested against *S. aureus* ATCC 29213, to assure bacterial viability throughout the assay. Since the MIC of some compounds have already been determined, i.e., **8** (8 µg/mL or 18 µM), **9** (256 µg/mL or >100 µM), **10** (>256 µg/mL or >100 µM), **12** (>64 µg/mL or >100 µM), **13** (8 µg/mL or 20 µM), **14** (>64 µg/mL or >100 µM), **16** (>64 µg/mL or >100 µM), **17** (2 µg/mL or 5 µM), **18** (4 µg/mL or 7 µM), and **19** (32 µg/mL or >100 µM) [16,17], the MIC of the remaining compounds was determined in this strain. Compounds **6** and **7** showed a MIC of 12.5 µM whereas **10** displayed an MIC > 100 µM. The concentrations used in both strains, as well as the percentages of biofilm inhibition, are shown in Table 2.

The results showed that all the tested compounds exhibited stronger inhibition of biofilm formation in *S. aureus* 272123 than in *S. aureus* ATCC 29213, with the exception of **13** and **19**. Moreover, **6**−**9, 12, 13, 16** and **19** showed higher inhibition of biofilm formation than reserpine in *S. aureus* 272123. Compounds **8, 9, 13,** and **19** were able to inhibit biofilm formation in both *S. aureus* strains tested. It should be noted that **8** and **9** displayed biofilm inhibition at very low concentrations. Compounds **6** and **16** could inhibit the efflux of ethidium bromide in *S. aureus* 272123, as well as biofilm formation, suggesting a possible link between the inhibition of efflux pumps and biofilm formation.

To study the QS inhibition by the compounds, three different systems were used: *Chromobacterium violaceum* CV026 (CV026), a sensor strain, and *Sphingomonas paucimobilis* Ezf 10-17 (EZF), a producer strain of acyl-homoserine lactones (AHL), which were inoculated as parallel lines; *Serratia marcescens* AS-1 and *Chromobacterium violaceum* wild-type 85 (wt85), both of which are AHL producers and were inoculated as single lines. The inhibition of QS was observed as a reduction in pigment production and measured in millimeters (mm). Promethazine (PMZ) was used as a positive control [45]. The results showed that there was only an inhibition of QS in the combination of EZF + CV026, with **8**, **10**, **13,** and **19** displaying discoloration of 30 ± 0.1 mm, 32 ± 0.8 mm, 31 ± 0.1 mm, and 42 ± 0.5 mm, respectively while PMZ displayed a discoloration of 41 ± 0.5 mm. The fact that **8** was also effective in the inhibition of EB accumulation in SE03 suggests a connection between these two phenomena, as bacteria of the *Chromobacterium* family have been described as possessing efflux systems of the RND family [46].

### 2.6. Cytotoxicity Assay

The last step to verify if the compounds are suitable for therapeutics is to determine their cytotoxicity in eukaryotic cells. The most promising compounds, **6**, **8**, **9**, **12**, and **17**–**19,** were tested for their cytotoxicity in mouse fibroblast cell line NIH/3T3. Doxorubicin was used as a positive control. The results are shown in Table 3 and the dose response curves are present in the Supplementary Data (Figure S2).

The results show that all the tested compounds displayed cytotoxicity against the tested cell line. However, except for the dimeric naphthopyranone **19**, the determined IC_50_ value is higher than the concentration needed to produce an antibacterial effect on the tested strains. The concentrations used to perform the efflux pump inhibition assay in SE03 were higher than their IC_50_ values in the case of **8** and **9**, meaning that these compounds must be studied at lower concentrations in order to determine their activity at a non-toxic concentration. Compound **12** also showed a lower IC_50_ value than the concentration tested for the biofilm formation assay, where the inhibition effect was greater than 50%. Most of the compounds (except **8**, **9**, **12**, and **19**) could have potential to be used in effective concentrations without toxicity. Previous studies have shown that **7** [47], **13**, and **14** [48] have also displayed cytotoxicity in other cell lines.

## 3. Materials and Methods

### 3.1. Compounds

All the compounds used in this study were previously isolated from natural sources. The indole alkaloids **1**–**5** and the meroditerpene **6** were isolated from *N. siamensis* [16]. The meroditerpenes **7** and **8** and the alkaloids **9** and **10** were isolated from *N. takakii* [19], and the meroditerpene **11** was isolated from *N. laciniosa* [34]. The amide-containing isochromenes **12** and **13** and the dimeric naphthopyranones **15**–**19** were isolated from *A. elegans* [17]. The purity of the compounds was evaluated by thin-layer chromatography prior to the assays, and the compounds presented the same NMR spectra as when they were first isolated.

### 3.2. Culture Media and Chemicals

The culture media used in these experiments were the following: cation-adjusted Mueller–Hinton broth (MHB II; Sigma-Aldrich, St. Louis, MO, USA and Biokar Diagnostics, Allone, Beauvais, France), Luria–Bertani broth (LB-B; Sigma, St. Louis, MO, USA), Tryptic Soy broth (TSB; Scharlau Chemie S. A., Barcelona, Spain), and Tryptic Soy agar (TSA; Biokar Diagnostics, Allone, Beauvais, France) were purchased. The modified Luria–Bertani agar (LB*-A) used for the quorum sensing (QS) inhibition assays was prepared in-house according to the formula: 1.0 g of yeast extract (Merck, Darmstadt, Germany), 10.0 g of tryptone (Biolab, Budapest, Hungary), 10.0 g of NaCl (Molar Chemicals, Halásztelek, Hungary), 1.0 g of K_2_HPO_4_ (Biolab, Budapest, Hungary), 0.3 g of MgSO_4_∙7H_2_O (Reanal, Budapest, Hungary), 5 mL of Fe-EDTA stock solution and 20.0 g of bacteriological agar (Molar Chemicals, Halásztelek, Hungary) per 1 L of media. *S. aureus* ATCC 29213 was purchased from ATCC (Manassas, VA, USA) and the mouse embryonic fibroblast cell line (NIH/3T3) was purchased from Sigma (Steinheim, Germany).

DMSO, 3-(4,5-dimethylthiazol-2-yl)-2,5-diphenyltetrazolium bromide (MTT), sodium dodecyl sulfate (SDS), phosphate-buffered saline (PBS; pH 7.4), EB, reserpine, CCCP, PMZ, ciprofloxacin, and crystal violet (CV) were purchased from Sigma-Aldrich Chemie GmbH (Steinheim, Germany). Doxorubicin was purchased from Teva Pharmaceuticals (Budapest, Hungary).

### 3.3. Bacterial Strains

As Gram-positive bacteria, *Staphylococcus aureus* ATCC 29213 and methicillin- and ofloxacin-resistant *Staphylococcus aureus* 272123 clinical isolates were used. As Gram-negative bacteria, the *acrA* gene-inactivated mutant *Salmonella enterica* serovar Typhimurium SL1344 (SE03) was investigated in this study.

For the QS tests, all the bacteria used were Gram-negative. The bacteria used were *Chromobacterium violaceum* wild type 85 (wt85), characterized by the AHL signal molecule-mediated production of the purple violacein pigment, capable of endogenous QS-signal molecule production (*N*-hexanoyl-l-HSL), *C. violaceum* CV026 (CV026), a Tn5 transposase-mutant, AHL-signal molecule indicator strain (produces purple violacein pigment in the presence of AHL), which is incapable of endogenous QS-signal molecule-production, but useful in the detection of external stimuli, *Sphingomonas paucimobilis* Ezf 10–17 (EZF), AHL-producing-strain (used with *C. violaceum* CV026), and *Serratia marcescens* AS-1, characterized by the AHL signal molecule-mediated production of the orange–red pigment prodigiosin (2-methyl-3-pentyl-6-methoxyprodigiosin), capable of endogenous QS-signal molecule production (*N*-hexanoyl-l-HSL), were applied [49].

### 3.4. Antibacterial Assay

The antibacterial activity was assessed by determination of the MIC of the compounds using the microdilution method, in a 96-well plate, according to the Clinical and Laboratory Standard Institute (CLSI) guidelines [50]. The media used was MHB II. The concentrations tested ranged from 100 µM to 0.195 µM. The MIC was determined by visual inspection. DMSO, in subinhibitory concentrations (1% *v/v*), was used as a solvent for the compounds.

### 3.5. Efflux Pump Inhibition Assay

Compounds **1**–**19** were evaluated for their ability to inhibit efflux pumps in SE03 and *S. aureus* 272123 strains, through real-time fluorimetry, monitoring the intracellular accumulation of EB, an efflux pump substrate. This was determined by the automated method using a CLARIOstar Plus plate reader (BMG Labtech, Ortenberg, Germany). Reserpine and CCCP were applied at 25 µM as positive controls, and the solvent DMSO was applied at 1% *v*/*v*. The bacterial strains were incubated in an appropriate culture media (TSB—*S. aureus* 272123; LB-B—SE03) at 37 °C until they reached an optical density (OD) between 0.4 and 0.6 at λ = 600 nm. The culture was centrifuged at 13,000× *g* for 3 min, and the pellet was washed and resuspended with PBS. The suspension was centrifuged again in the same conditions and resuspended in PBS. The compounds were applied at 50 µM in a solution of a non-toxic concentration of EB (1 µg/mL) in PBS. Then, 50 µL of this solution were transferred into a 96-well black microtiter plate (Greiner Bio-One Hungary Kft, Mosonmagyaróvár, Fertősor, Hungary), and 50 µL of bacterial suspension (OD_600_ 0.4–0.6) were added to each well. The plates were placed into the CLARIOstar plate reader, and the fluorescence was monitored at excitation and emission wavelengths of 530 nm and 600 nm every minute for one hour on a real-time basis. From the real-time data, the activity of the compounds, namely the RFI of the last time point (minute 60) of the EB accumulation assay, was calculated according to the following formula:

*RFI* = (*RF*_*treated*_ − *RF*_*untreated*_)/*RF*_*untreated*_ (1)

where *RF_treated_* is the relative fluorescence (RF) at the last time point of EB accumulation curve in the presence of the compound, and *RF_untreated_* is the RF at the last time point of the EB accumulation curve of the untreated control, having only the solvent (DMSO) control. The accumulation curves were designed using Microsoft Excel 365^®^ (Microsoft Corporation, Redmond, WA, USA).

### 3.6. Docking Studies

The crystal structures of the AcrB (PDB: 4DX5) [51], AcrA (PDB: 2F1M) [52], and TolC (PDB: 1EK9) [53] portions of the AcrAB-TolC bacterial efflux system, downloaded from the protein databank (PDB) [54], were used for this study. The structures of the known AcrAB-TolC inhibitors D13-9001, doxorubicin, MBX-3132, minocycline, and phenyl-arginyl-β-naphthylamide, along with the structures of the tested compounds were drawn with ChemDraw 17 (PerkinElmer Informatics, Waltham, MA, USA) and minimized using ArgusLab 4.0.1 (Mark Thompson and Planaria Software LLC, Seattle, WA, U.S.A.). Docking was carried out using AutoDock Vina 0.8 (Scripps, La Jolla, CA, USA) [55], in the sites described in [40,41]. Since the crystal structure of NorA efflux pump is not available, a homology model was prepared. The model was generated using the Swiss Model server [56] and the sequence was deposited in Uniprot (Q5HHX4) [57], using the EmrD pump from *Escherichia coli* (PDB: 2GFP) as the homolog, as described previously [42]. The sequence similarity was 0.28, the coverage was 0.91 and the sequence identity 17.33%. The top nine poses were collected for each molecule and the lowest docking score value was associated with the most favorable binding conformation. PyMol 0.99 (Schrödinger, New York, NY, USA) was used for molecular visualization [58].

Compounds **1**–**19** were docked into sites already described in the literature. For AcrB, the sites studied were the substrate-binding site (SBS) and the hydrophobic trap (HT) [40]; for AcrA, the docking studies were performed in the helical hairpin (HH) and the lipoyl domains (LD) [41]; for TolC, the site considered comprised the lysine residues that interact with the 3,3′-dithiobis(sulfosuccinimidyl propionate) bifunctional crosslinker [41]. Concerning the NorA homology model, the sites used for the docking studies were the binding core region (BCR) and the cytoplasmic side (CS), as described in [42]. The position and dimensions of the grid used for each site are present in Table 4.

### 3.7. Inhibition of Biofilm Formation

Compounds **6**–**10**, **12**–**14**, and **16**–**19** were tested for their ability to inhibit the formation of biofilm. The bacterial strains used were the Gram-positive *S. aureus* ATCC 25923 and *S. aureus* 272123. The detection of the biofilm formation was possible with the use of the dye crystal violet (CV; 0.1% *v/v*). The initial inoculum was incubated in TSB overnight, and then diluted to an OD_600_ of 0.1. Then, the bacterial suspension was added to 96-well microtiter plates and the compounds were added at a concentration of ½ MIC, and for compounds whose MIC is higher than 100 µM, a concentration of 100 µM was used. The final volume in each well was 200 µL. Reserpine was used as the positive control, as it was the same compound used in the efflux pump inhibition assay and it has shown activity in the inhibition of biofilm formation in *S. aureus* strains [43]. The plates were incubated at 30 °C for 48 h, with gentle stirring (100 rpm). After this incubation period, the TSB medium was discarded, and the plates were washed with tap water to remove unattached cells. Afterwards, 200 µL of a 0.1% *v/v* CV solution were added to the wells and incubated for 15 min at room temperature. Then, the CV solution was removed from the wells, the plates were washed again with tap water, and 200 µL of a 70% ethanolic solution were added to the wells. The biofilm formation was determined by measuring the OD_600_ using a Multiscan EX ELISA plate reader (Thermo Labsystems, Cheshire, WA, USA). The anti-biofilm effect of the compounds was expressed as the percentage (%) of a decrease in biofilm formation.

### 3.8. Quorum-Sensing Assay

The QS inhibitory effect of the compounds was examined on the EZF and the sensor CV026 strains, on the wt85 strain, and on *S. marcescens*, for **6**–**10**, **12**–**14**, and **16**–**19**. The method used was the parallel inoculation method, where pair combinations of the used sensor strain CV026 and the AHL-producing strain EZF were inoculated directly onto the LB*-A agar surface in parallel, at an approximate distance of 5 mm from each other. *S. marcescens* AS-1 and wt85 were inoculated as a single line. Filter paper disks (7 mm in diameter) were placed on the center of the inoculated line(s) and impregnated with 8 µL of a solution of 10 mM of the compounds. PMZ was used as the positive control, as previous results have demonstrated its activity as a QS inhibitor [45]. The agar plates were incubated at room temperature (20 °C) for 24–48 h. The QS inhibition was accessed visually, through the inhibition of pigment production. The discolored, but intact, bacterial colonies were measured with a ruler [45,49,59].

### 3.9. Cytotoxicity Assay

A 10.0 mM stock solution of each compound was prepared in DMSO. All stock solutions were stored at −80 °C and freshly diluted on the day of the experiment in fresh cell culture medium (ensuring that DMSO did not exceed 0.1% DMSO concentration of the exposure media).

Mouse fibroblasts (NIH/3T3, ATCC CRL-1658TM) were cultivated in Dulbecco’s Modified Eagle Medium (DMEM, Gibco 52100-039) and supplemented with 10% heat-inactivated fetal bovine serum (Biowest, VWR International Kft, Debrecen, Hungary), 2 mM of l-glutamine, 1 mM sodium pyruvate, 100 U/L and 10 mg/L penicillin/streptomycin mixture (Sigma-Aldrich Chemie GmbH, Steinheim, Germany), respectively, and 0.1% nystatin (8.3 g/L in ethylene glycol). The adherent cells were detached using a combination of 0.25% Trypsin–Versene (EDTA) solution for 5 min at 37 °C. Before each cytotoxicity assay using this cell line, cells were seeded in untreated 96-well flat-bottom microtiter plates, following a 4 h incubation period in a humidified atmosphere (5% CO_2_, 95% air) at 37 °C [60].

The cytotoxicity of **6**, **8**, **9**, **12**, and **17**–**19** was assessed in NIH/3T3 cells, using the MTT assay. Prior to the assay, the cells were seeded for 4 h using 1 × 10^4^ cells/well. The compounds were added by two-fold serial dilutions to the cells distributed into 96-well flat bottom microtiter plates starting with 100 μM. The plates were incubated for 24 h, after which a solution of MTT in PBS was added to each well and incubated for another 4 h. After this, 100 μL of SDS (10% in a 0.01 M HCl solution) were added to each well and incubated overnight at 37 °C. Doxorubicin was used as the positive control. Cell growth was determined in quadruplicate by measuring OD at λ = 540 nm (reference 630 nm) in a Multiscan EX ELISA reader (Thermo Labsystems, Cheshire, WA, USA). The percentage of inhibition of cell growth was determined according to the equation:

(2) $$100-\left(\frac{{\mathrm{OD}}_{\mathrm{sample}}-{\mathrm{OD}}_{\mathrm{medium}\;\mathrm{control}}}{{\mathrm{OD}}_{\mathrm{cell}\;\mathrm{control}}-{\mathrm{OD}}_{\mathrm{medium}\;\mathrm{control}}}\right)\times 100$$

The results were expressed as the mean ± standard deviation (SD), and the IC_50_ values were obtained by best fitting the dose-dependent inhibition curves in GraphPad Prism 5.03 for Windows software.

## 4. Conclusions

The results presented herein reinforce the importance of the marine environment as a source of bioactive compounds with remarkably varied applications. From a library of 19 compounds, nine compounds, i.e., two meroditerpenes (**6** and **8**), two alkaloids (**9** and **10**), two mycotoxins (**13** and **14**), and three dimeric naphthopyranones (**17**–**19**) exhibited antibacterial activity in an oxacillin- and methicillin-resistant strain of *S. aureus.*

In terms of the efflux inhibition, a meroditerpene **6**, and a dimeric naphthopyranone **16**, were effective in the Gram-positive model, whereas a meroditerpene **8**, an alkaloid **9**, and an amide-containing isochromene **12** were effective in the Gram-negative model. These compounds were considered promising and were tested for their potential as inhibitors of biofilm formation and QS. It was observed that **8**, **9**, **13**, and **19** exhibited a sharp decrease in biofilm formation in both *S. aureus* strains tested. Additionally, for the resistant *S. aureus* strain, **6**, **7**, **12**, and **16** were also effective in the reduction of biofilm formation. Furthermore, **8**, **10**, **13**, and **19** showed inhibition of QS. However, **8**, **9**, **12**, and **19** displayed cytotoxicity in the tested cell line, inhibiting more than 50% of cell growth at the concentration they displayed efficacy at in the various assays. On the other hand, **6**, **17,** and **18** displayed relevant activity at non-cytotoxic concentrations for the tested cell line, highlighting meroditerpene chevalone C (**6**) as a promising efflux pump inhibitor and vioxanthin (**17**) and viomellein (**18**) as antimicrobials with a biofilm formation inhibitory mechanism.

It can be concluded that, except for the tryptoquivalines, at least one compound from each class was effective in the activities tested herein. This shows that the marine environment is indeed a source of useful compounds for pressing public health problems, such as antimicrobial resistance.

The fact that these compounds present cytotoxicity should be regarded as an opportunity to focus on a future work in the synthesis of derivatives with improved toxicological profiles while retaining their activities. This poses a medicinal chemistry challenge, as both the total synthesis of such complex molecules and the isolation of enough quantity of compounds to perform chemical modifications are very arduous processes. However, these compounds may inspire new substitution patterns in simpler molecules, such as xanthones, which have been described as efflux pump inhibitors and are structurally similar to compounds herein presented, such as the dimeric naphthopyranones **17** and **18**. Future work to be developed within this scope may also involve more thorough studies into the efflux mechanisms, as an attempt to decipher if the inhibition of the efflux of ethidium bromide is, in fact, related to the efflux of efflux pumps, and which efflux pump is specifically being inhibited.

## Figures and Tables

**Table 1:** Minimum inhibitory concentration (MIC) of **1**–**19**.

Compounds	MIC (µM)	
	*S. aureus* 272123	SE03
**1**	>100	>100
**2**	>100	>100
**3**	>100	>100
**4**	>100	>100
**5**	>100	>100
**6**	25	>100
**7**	>100	>100
**8**	12.5	>100
**9**	12.5	>100
**10**	100	>100
**11**	>100	>100
**12**	>100	>100
**13**	50	>100
**14**	50	>100
**15**	>100	>100
**16**	>100	>100
**17**	6.25	>100
**18**	6.25	>100
**19**	100	>100
Ciprofloxacin	12.5	6.25

SE03: *S. enterica* serovar Typhimurium SL1344.

**Table 2:** Biofilm inhibition by **6–10**, **12**–**14** and **16**–**19**.

Compounds	*S. aureus* ATCC 29213		*S. aureus* 272123	
	Concentration (µM)	Biofilm Inhibition ± SD (%)	Concentration (µM)	Biofilm Inhibition ± SD (%)
**6**	6.25	0	12.5	78.48 ± 7.97
**7**	6.25	0.13 ± 0.08	100	85.55 ± 0.61
**8**	9	72.31 ± 2.29	6.25	93.41 ± 0.91
**9**	100	63.18 ± 2.42	6.25	93.47 ± 2.22
**10**	100	0	50	0
**12**	100	0	100	80.78 ± 5.27
**13**	10	87.92 ± 1.55	25	97.89 ± 0.94
**14**	100	0	25	0
**16**	100	0	100	92.58 ± 1.97
**17**	2.5	0	3.13	4.31 ± 2.48
**18**	3.5	2.47 ± 1.66	3.13	6.17 ± 0.75
**19**	100	95.73 ± 0.45	50	84.23 ± 2.94
Reserpine	25	22.29 ± 5.10	25	72.1 ± 4.24

SD: Standard deviation. The compounds that displayed a MIC > 100 µM were tested at 100 µM.

**Table 3:** IC_50_ values (µM) of **6**, **8**, **9**, **12**, and **17**–**19** in cytotoxicity assay against NIH/3T3.

Compounds	IC_50_ (µM)
**6**	30.95 ± 0.13
**8**	25.02 ± 2.37
**9**	16.74 ± 1.40
**12**	80.02 ± 3.66
**17**	34.50 ± 3.14
**18**	16.71 ± 1.52
**19**	32.44 ± 3.22
Doxorubicin	12.05 ± 0.81

**Table 4:** Position and dimensions of the grid used to perform the docking studies.

Structure	Site	Position			Dimension		
		X	Y	Z	X	Y	Z
AcrA (2F1M)	HH	27.4205	14.1758	175.9638	15.9628	12.8742	17.6756
	LD	26.8634	−2.5985	207.5824	15.9628	11.6319	27.9448
AcrB (4DX5)	SBS	24.3266	−32.1670	−7.0000	18.4129	26.7613	20.1435
	HT	20.8792	17.7378	−7.0708	14.5855	17.7378	15.3042
TolC (1EK9)		−7.8482	84.1409	63.4236	39.5596	29.8075	15.9794
NorA	BCR	−4.3807	−19.3774	20.8856	14.5855	17.2122	20.5459
	CS	−9.2889	−27.7277	42.4691	14.5855	17.2122	17.3139

SBS: Substrate-binding site; HT: Hydrophobic trap; HH: Helical hairpin; LD: Lipoyl domain; BCR: Binding core region; CS: Cytoplasmic side.

## Supplementary Materials

The following are available online at https://www.mdpi.com/article/10.3390/md19090475/s1, Table S1. Relative fluorescence index (RFI) of **1**–**19**; Table S2. Docking results for **1**–**19** in bacterial efflux pumps; Figure S1: Fluorescence studies on compound **7**; Figure S2. Dose-response curves for the tested compounds.
