# Supplementary material for: Metabolites from Marine-Derived Fungi as Potential Antimicrobial Adjuvants
Source: Mar Drugs. 2021 Aug 25;19(9):475. doi: 10.3390/md19090475 (PMC8470461; doi:10.3390/md19090475)
Supplement: Supplementary file 1 [file marinedrugs-19-00475-s001.zip › marinedrugs-1314441-supplementary.pdf]

Supplementary Data

Metabolites from Marine-Derived Fungi as Potential Antimicrobial Adjuvants

Fernando Durães [1,2], Nikoletta Szemerédi [3], Madalena Pinto [1,2], Decha Kumla [2,4], Anake Kijjoa [2,4], Gabriella Spengler [3*] and Emília Sousa [1,2*]

1 Laboratory of Organic and Pharmaceutical Chemistry, Department of Chemical Sciences, Faculty of Pharmacy, University of Porto, Rua de Jorge Viterbo Ferreira, 228, 4050-313 Porto, Portugal; fduraes5@gmail.com (F. D.); madalena@ff.up.pt (M.P.).
2 CIIMAR – Interdisciplinary Centre of Marine and Environmental Research, University of Porto, Novo Edifício do Terminal de Cruzeiros do Porto de Leixões, Avenida General Norton de Matos, S/N, 4450-208 Matosinhos, Portugal; ankijjoa@icbas.up.pt (A. K.)
3 Department of Medical Microbiology, Albert Szent-Györgyi Health Center and Faculty of Medicine, University of Szeged, Semmelweis utca 6, 6725 Szeged, Hungary; szemeredi.nikoletta@med.u-szeged.hu (N. S.).
4 *ICBAS* – Institute of Biomedical Sciences Abel Salazar, Universidade do Porto, Rua de Jorge Viterbo Ferreira 228, 4050-313 Porto, Portugal;
* Correspondence: spengler.gabriella@med.u-szeged.hu (G. S.); esousa@ff.up.pt (E. S.).

Efflux pump inhibition assay

Table S1. Relative fluorescence index (RFI) of **1-19**.

Compound	*S. aureus* 272123		SE03	
	Concentration (µM)	RFI ± SD	Concentration (µM)	RFI ± SD
**1**	50	-0.01 ± 0.04	50	0.03 ± 0.06
**2**	50	-0.21 ± 0.05	50	-0.15 ± 0.04
**3**	50	0.15 ± 0.05	50	0.07 ± 0.03
**4**	50	-0.02 ± 0.01	50	0.09 ± 0.04
**5**	50	0.07 ± 0.02	50	0.08 ± 0.02
**6**	8.33	0.99 ± 0.11	50	-0.02 ± 0.01
**7**	50	1.43 ± 0.20	50	0.22 ± 0.01
**8**	4.17	0.55 ± 0.01	50	3.38 ± 0.10
**9**	4.17	0.43 ± 0.07	50	2.38 ± 0.12
**10**	33.3	0.26 ± 0.06	50	0.22 ± 0.03
**11**	50	0.41 ± 0.02	50	-0.14 ± 0.01
**12**	50	0.29 ± 0.01	50	0.45 ± 0.02
**13**	16.67	-0.37 ± 0.02	50	-0.12 ± 0.02
**14**	16.67	-0.19 ± 0.01	50	-0.05 ± 0.01
**15**	50	-0.17 ± 0.03	50	-0.03 ± 0.01
**16**	50	1.09 ± 0.09	50	-0.11 ± 0.01
**17**	2.08	-0.07 ± 0.03	50	-0.72 ± 0.01
**18**	2.08	0.31 ± 0.09	50	-0.63 ± 0.01
**19**	33.3	-0.27 ± 0.13	50	-0.36 ± 0.01
**Reserpine**	25	0.59 ± 0.03	-	-
**CCCP**	-	-	25	0.33 ± 0.04

SE03: *S. enterica* serovar Typhimurium SL1344; **SD**: Standard deviation; **CCCP**: carbonyl cyanide 3-chlorophenylhydrazone; The compounds that displayed a MIC > 100 µM were tested at 50 µM.

Fluorescence assay

In order to study if the fluorescence of the compounds was an interference in the real-time ethidium bromide accumulation assay, the compounds that displayed higher fluorescence than the controls in the beginning of the assay were investigated. This was only the case for compound **7**, as compounds **6**, **8**, **9**, **12**, and **16** showed relative fluorescence index approximate to the ones observed for DMSO and the positive controls.

As such, an assay was developed, where the fluorescence of a solution of compound **7** in PBS was tested against a solution of compound **7** and 1 µg/ml EB and a solution of 1 µg/ml EB. For the fluorescence of the compound to not be considered a hindrance of the assay, its fluorescence in combination with EB curve must be similar to that of EB alone, and the curve of the compound alone must be close to zero. These results can be seen in Figure S1.

Compound **7** presents an erratic fluorescence curve in combination with EB, which surpasses the RFI of EB alone, even though its fluorescence alone is constant and close to zero. Consequently, the results obtained with this compound for the efflux pump inhibition assay were not considered.

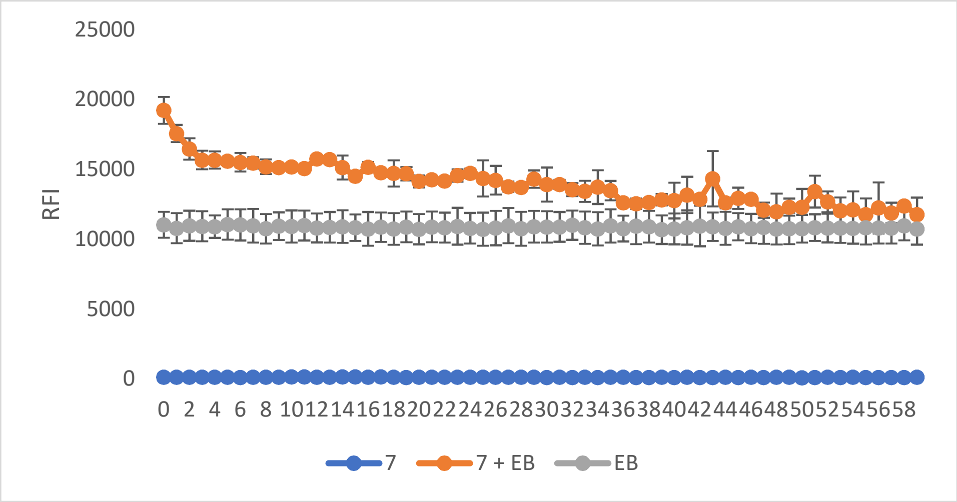

Figure S1. Curves relative to the fluorescence studies on compound **7**, compound **7** in combination with EB, and EB alone.

Docking studies

Analysis of the docking scores in Table S2 showed that, within the AcrAB-TolC efflux system, all the compounds are predicted to display more affinity towards the AcrB portion, and particularly against the substrate-binding site (SBS) (except **14** and **17**), similar to the positive controls. On the other hand, the hydrophobic trap (HT) of AcrB is the site with the least predicted affinity. The periplasmic adaptor protein AcrA is a portion for which the compounds are predicted to globally display less affinity. In the NorA homology model, there is a clear preference towards the cytoplasmic side (CS), which is in line with the results already described for the positive control reserpine, a known NorA inhibitor.

It can be also observed that most of the compounds present a docking score resembles that of the compounds already described to present efflux pump inhibition in the portions of the AcrAB-TolC efflux system and in the cytoplasmic side of NorA. Noteworthy is also the fact that some compounds exhibited high predicted binding energy, particularly in the NorA homology model and in the hydrophobic trap of AcrB. This observation may be related to the fact that these compounds are very bulky and present rigid conformations and may not fit in the sites defined as potential binding sites, which were used for the docking studies. However, given their favorable results with the SBS of AcrB and with the CS of NorA, and the similarity of their docking scores to those obtained for xanthones that exhibited the inhibition of bacterial efflux pumps, all the studied compounds were tested for this activity.

Table S2. Docking results for **1–19** in bacterial efflux pumps.

Compound	Docking Score (kcal/mol)						
	AcrB		AcrA		TolC	NorA	
	SBS	HT	HH	LD		BCR	CS
**1**	−9.2	−3.2	−7.9	−6.5	−8.5	0.1	−6.7
**2**	−9.6	17.4	−7.0	−6.2	−8.4	−0.3	−6.9
**3**	−9.3	1.7	−6.7	−6.1	−8.6	1.1	−6.9
**4**	−9.1	25.2	−6.9	−5.8	−8.5	1.7	−6.6
**5**	−8.9	0.7	−6.8	−6.2	−8.3	0.4	−6.8
**6**	−9.2	22.4	−6.7	−5.6	−9.0	−3.1	−6.7
**7**	−9.4	34.9	−6.9	−6.2	−9.4	−3.6	−7.4
**8**	−8.4	11.6	−6.8	−6.2	−8.4	−1.7	−5.8
**9**	−8.3	2.9	−6.4	−5.5	−7.5	1.2	−6.7
**10**	−8.3	26.1	−6.4	−6.4	−7.7	2.8	−6.5

**11**	−8.5	11.0	−6.3	−6.2	−8.6	−2.3	−7.6
**12**	−8.0	−2.5	−6.3	−5.8	−7.5	−7.4	−7.4
**13**	−8.0	−5.8	−6.3	−5.6	−7.4	−7.5	−6.4
**14**	−8.0	−2.1	−6.8	−6.4	−8.0	−7.5	−5.9
**15**	−9.1	15.1	−6.8	−6.9	−8.5	7.3	19.8
**16**	−9.7	31.6	−6.7	−5.7	−8.1	4.8	28.0
**17**	−9.0	7.5	−6.2	−6.0	−9.3	3.6	−4.2
**18**	−9.3	40.5	−5.9	−5.9	−8.4	2.5	4.4
**19**	−10.9	29.9	−5.8	−5.2	−9.2	7.7	−5.3
**Reserpine**	−8.7	10.9	5.6	4.6	−7.5	1.0	−4.6
**PAβN**	−7.1	−4.7	−5.8	−4.9	−7.1	−9.4	−5.3
**D13-9001**	−9.7	26.5	−6.2	−5.1	−7.4	-	-
**Doxorubicin**	−8.9	15.4	−7.2	−5.6	−7.2	-	-
**MBX-3132**	−7.9	2.9	−7.9	−6.2	−7.7	-	-
**Minocycline**	−8.7	26.7	−6.2	−5.4	−7.7	-	-

SBS: Substrate-binding site; **HT**: Hydrophobic trap; **HH**: Helical hairpin; **LD**: Lipoyl domain; **BCR**: Binding core region; **CS**: Cytoplasmic side.

Since the compounds can be grouped, according to their structural similarity (**1** – **5**, **6** – **8** and **11**, **9** and **10**, **12** – **14**, **15** and **16**, and **17** – **19**), structure-activity relationships could possibly be established, and the visualization of these compounds within the predicted binding site (section 2.4) in the target may help shed light on possible future synthetic work.

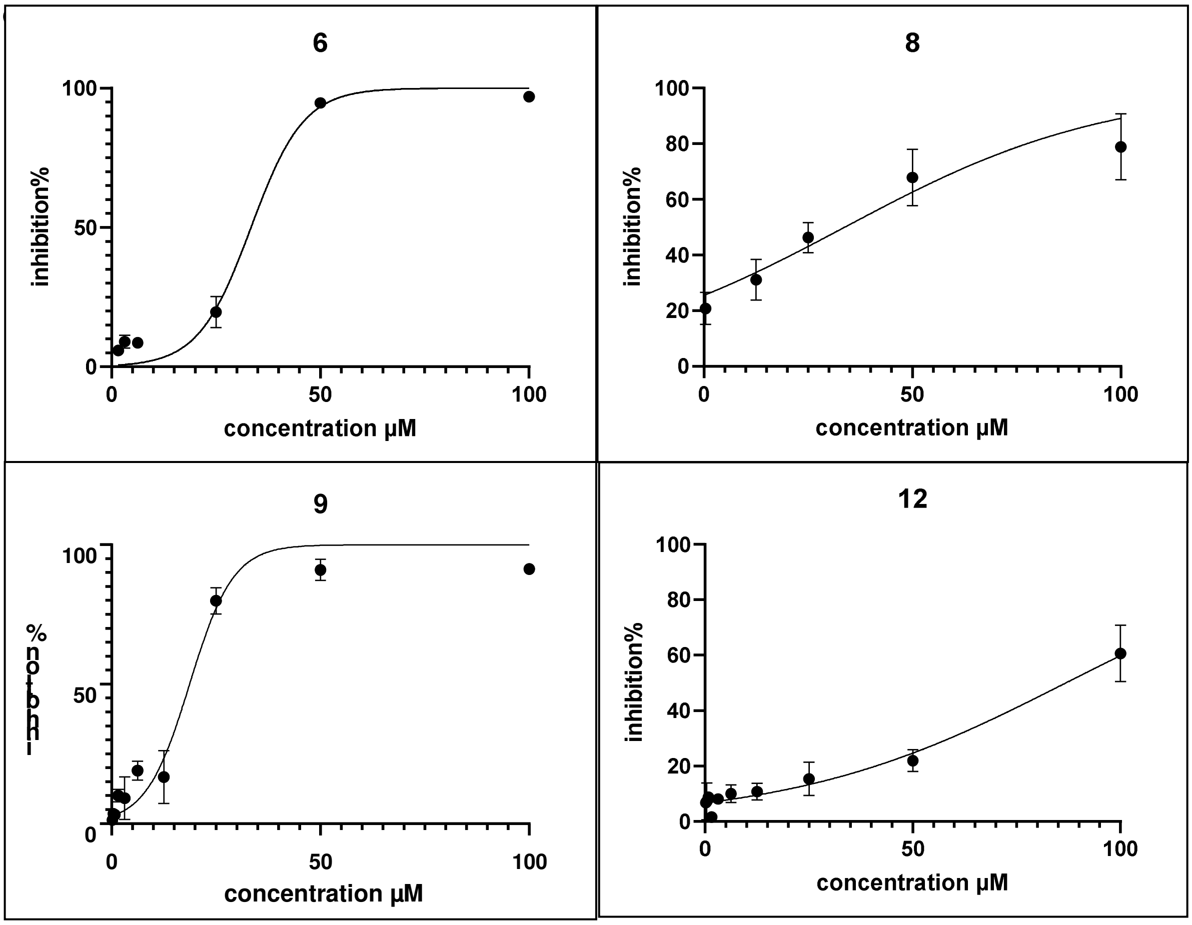

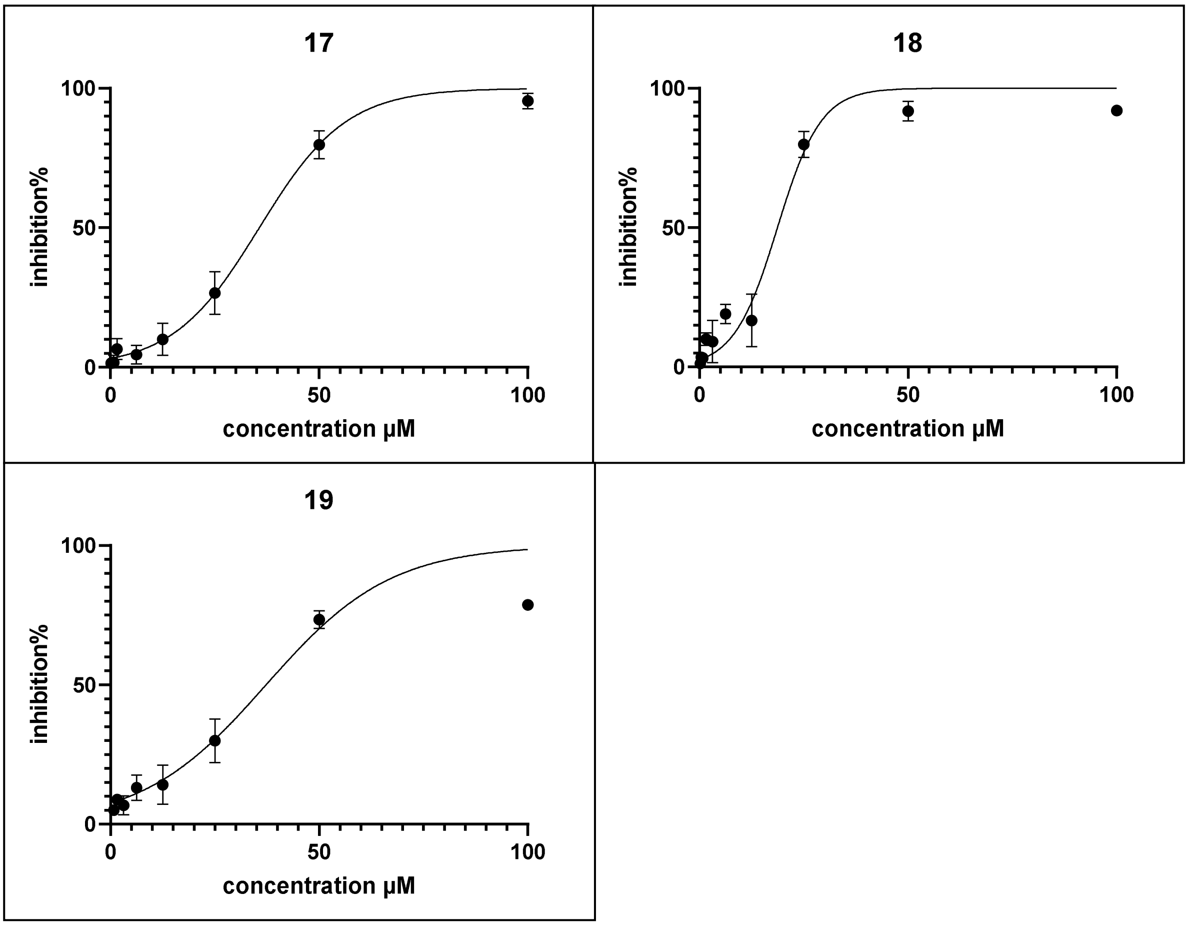

Figure S2. Dose-response curves for the tested compounds.
